# Effect of Chronic Restraint Stress on Human Colorectal Carcinoma Growth in Mice

**DOI:** 10.1371/journal.pone.0061435

**Published:** 2013-04-09

**Authors:** Qiang Lin, Feifei Wang, Rong Yang, Xinmin Zheng, Huibao Gao, Ping Zhang

**Affiliations:** 1 Department of Biochemistry and Molecular Cell Biology, Institute of Medical Science, Shanghai Jiaotong University School of Medicine, Shanghai, P.R. China; 2 Department of Molecular Biology and Genetics, Cornell University, Ithaca, New York, United States of America; Harvard Medical School, United States of America

## Abstract

Stress alters immunological and neuroendocrinological functions. An increasing number of studies indicate that chronic stress can accelerate tumor growth, but its role in colorectal carcinoma (CRC) progression is not well understood. The aim of this study is to investigate the effects of chronic restraint stress (CRS) on CRC cell growth in nude mice and the possible underlying mechanisms. In this study, we showed that CRS increased the levels of plasma catecholamines including epinephrine (E) and norepinephrine (NE), and stimulated the growth of CRC cell-derived tumors in vivo. Treatment with the adrenoceptor (AR) antagonists phentolamine (PHE, α-AR antagonist) and propranolol (PRO, β-AR antagonist) significantly inhibited the CRS-enhanced CRC cell growth in nude mice. In addition, the stress hormones E and NE remarkably enhanced CRC cell proliferation and viability in culture, as well as tumor growth in vivo. These effects were antagonized by the AR antagonists PHE and PRO, indicating that the stress hormone-induced CRC cell proliferation is AR dependent. We also observed that the β-AR antagonists atenolol (ATE, β1- AR antagonist) and ICI 118,551 (ICI, β2- AR antagonist) inhibited tumor cell proliferation and decreased the stress hormone-induced phosphorylation of extracellular signal-regulated kinases-1/2 (ERK1/2) in vitro and in vivo. The ERK1/2 inhibitor U0126 also blocked the function of the stress hormone, suggesting the involvement of ERK1/2 in the tumor-promoting effect of CRS. We conclude that CRS promotes CRC xenograft tumor growth in nude mice by stimulating CRC cell proliferation through the AR signaling-dependent activation of ERK1/2.

## Introduction

Colorectal carcinoma (CRC) represents one of the most common types of cancer worldwide [1]. The development of CRC typically results from both genetic and environmental factors. Stress, as one of the environmental factors, is linked to the occurrence and progression of CRC [2]. The stress response is a complex process that can protect the organisms from the potential threat and initiate a cascade of reactions, including activation of the sympathetic nervous system (SNS) and the hypothalamic-pituitary-adrenal (HPA) axis [3], [4], [5].

The catecholamines epinephrine (E) and norepinephrine (NE), which are known as the classic stress hormones, are synthesized by the adrenal medulla and the nerves of the SNS. Both E and NE are elevated in individuals with acute or chronic stress [6], [7]. Once chronically elevated, these stress hormones have been shown to increase tumor cell proliferation [8], [9], [10], adhesion [11], migration [12], [13], and invasion [14].

Epidemiological studies have demonstrated that distress in cancer patients might be associated with increased cancer progression [15], [16]. Conversely, social support has been shown to lengthen cancer patient survival [17], [18]. Experimental animal studies have demonstrated the effects of stress on tumor growth. For instance, immobilization stress has been shown to increase the incidence and tumor growth of chemically induced liver cancer in rats [19]. Stress has also been shown to promote mammary tumor development [20], [21] and ovarian cancer growth and angiogenesis [22] in animal models.

Previous studies have demonstrated that the stress hormones E and NE, via their specific adrenoceptors (ARs), promote cancer cell proliferation [22], [23], [24], [25], [26], [27], migration, and invasion [12], [28], as well as tumor growth, by enhancing angiogenesis [22]. However, if chronic stress can enhance CRC tumor growth, the underlying mechanism remains to be determined.

In this work, we studied the effect of CRS on CRC cell growth in nude mice and investigated the underlying mechanisms. The present study provides additional insights into understanding the pathogenesis of CRC, particularly in relation to chronic stress.

## Materials and Methods

### Drugs and antibodies

Epinephrine (E), norepinephrine (NE), corticosterone (CORT), isoproterenol (ISO, nonselective β-AR agonist), phentolamine (PHE, nonselective α-AR antagonist), propranolol (PRO, nonselective β-AR antagonist), atenolol (ATE, specific β1-AR antagonist), ICI 118,551 (ICI, specific β2-AR antagonist) and U0126 (specific ERK1/2 inhibitor) were purchased from Sigma (St. Louis, MO, USA). The following antibodies were used: monoclonal antibody specific for β1-AR from Bioworld Technology (St. Louis Park, MN, USA), polyclonal antibody specific for β2-AR from AbCam Biochemicals (Cambridge, UK), total ERK1/2 and phospho-ERK1/2 antibodies from R&D Systems (Minneapolis, MN, USA), anti-PCNA antibody from Proteintech Group (Chicago, IL, USA), anti-Ki-67 antibody from Santa Cruz Biotechnology (Santa Cruz, CA, USA) and anti-GAPDH antibody (glyceraldehyde-3-phosphate dehydrogenase) from Cell Signaling Technology (Danvers, MA, USA).

### Cell culture, proliferation and viability assays

The human CRC HT29, SW116 and LS174T cell lines were obtained from American Type Culture Collection (Manassas, VA, USA). HT29 cells were cultured in McCoy's 5A medium from Gibco, Life Technologies (Grand Island, NY, USA), and the other cell lines were grown in RPMI-1640 from Gibco, Life Technologies (Grand Island, NY, USA). For cell growth, the media were supplemented with 10% fetal bovine serum (FBS) and antibiotics (100 U/ml penicillin, 100 µg/ml streptomycin). Cells were maintained in an incubator at 37°C and in a humidified atmosphere containing 5% CO_2_. For the experiments on cell proliferation and viability, 5×10^3^ cells per well were seeded in 96-well plates. The medium was supplemented with antibiotics plus 1% FBS for cell attachment, and then, the cells were starved in serum-free medium for another 12 hours to synchronize the cell cycle. Different concentrations of E or NE (0, 0.1, 1, 10 µM) were then incubated with the CRC cells for 24 hours to study the growth-promoting effect of the hormones. To examine the effects of various inhibitors, the cells were pretreated with or without PHE (50 µM), PRO (50 µM), ATE (50 µM), ICI (50 µM) or U0126 (20 µM) for 45 min prior to E or NE treatment. Cell proliferation was indicated by the amount of DNA synthesis measured with the BrdU incorporation assay kit from Roche Applied Science (Nutley, NJ, USA), according to the manufacturer's instructions. Briefly, cells were labeled with 10 µl/well BrdU and incubated at 37°C for 4 hours. After removal of the labeling medium, the cells were fixed and probed with the anti-BrdU monoclonal antibody at 25°C for 2 hours and its substrate tetramethyl-benzidine (TMB) at 25°C for 30 min. After removal of the unconjugated antibody, the cells were rinsed 3 times with the washing solution and treated with 300 µl/well substrate solution. After color development, 1 M H_2_SO_4_ was added (25 µl/well) to stop the substrate reaction, and the absorbance of each sample was measured in an enzyme-linked immunosorbent assay (ELISA) microplate reader at 450 nm. A blank was run in each experiment to account for nonspecific binding to the anti-BrdU antibody. The value from the nonspecific binding was subtracted from all other values. For cell viability, a Cell Counting Kit-8 (CCK-8) from Dojindo Laboratories (Kumamoto, Japan) was used to determine CRC cell survival after E/NE treatments, according to the manufacturer's instructions. Cell counting was performed based on trypan blue exclusion.

### CRS model

Ten- to 12-week-old female athymic BALB/c nude mice were obtained from the Animal Center of the Chinese Academy of Science and were maintained in the division of laboratory animal science at Shanghai Jiaotong University School of Medicine (SJUSM). The mice were habituated to vivarium conditions for 1 week before initiating the stress procedures. We utilized a CRS model procedure established in our laboratory and others [22], [29], [30], [31], [32]. Briefly, the mice were restrained horizontally in 50 ml conical centrifuge tubes that were drilled with holes to allow for ample ventilation; the tubes were small enough to restrain mice so that they were able to breathe but unable to move freely. With this system, the mice were stressed daily for 6 h between 9:00 a.m.–3:00 p.m. During the rest period, the mice were provided with food and water ad libitum. We used only female nude mice because male nude mice were believed to be already under stress from caging with their mates [26]. All efforts were made to minimize the number of animals used and their suffering.

### Tumor induction

Cells were harvested from subconfluent cultures by a brief exposure to 0.25% trypsin in 0.02% EDTA, suspended in phosphate-buffered saline (PBS) and tested for >95% viability by trypan blue exclusion. The mice were randomized to two treatment groups: (1) no-stress control group and (2) CRS group. A total of 2×10^6^ HT29 or SW116 cells were implanted subcutaneously (s.c.) into the right flanks of nude mice 7 d after starting the stress treatment under sterile conditions. The stress procedure continued for another 21 d. Body weights were monitored throughout the experiment. The mice were sacrificed 21 d after tumor cell injection, and the tumors were harvested and weighed.

For the stress hormone E stimulation, 4 d after HT29 cell (2×10^6^) injection, we subcutaneously inserted microosmotic pumps (Alzet model 1002, Durect, Cupertino, CA,USA) filled with PBS containing different concentrations of E (0, 0.02 mg/kg, and 2 mg/kg) into the backs of nude mice for 2 weeks.

For the adrenoceptor (AR)-blockade, the nude mice were randomized to four treatment groups: (1) control PBS, (2) control PHE (2 mg/kg/d) + PRO (2 mg/kg/d), (3) CRS PBS and (4) CRS PHE (2 mg/kg/d) + PRO (2 mg/kg/d). The microosmotic pumps containing a combination of PHE and PRO, as well as 0.2% ascorbic acid as a preservative, were inserted into the nape of the neck 7 d before stress initiation [22]. HT29 cells were inoculated s.c. into the right flanks of nude mice 7 d after initiating stress. The mice were sacrificed 14 d after tumor cell injection.

To further determine the involvement of β-AR in the tumor-induction process, 7 d after HT29 cell injection, the pumps were filled with PBS containing E (0.02 mg/kg), a combination of E and ATE (5 mg/kg), or a combination of E and ICI (5 mg/kg), as well as ascorbic acid as a preservative, into the backs of nude mice for 2 weeks. The pumps of the control group were filled with PBS and ascorbic acid. All tumors from the various treatment groups were excised and fixed in 10% formalin. They were then either paraffin embedded or frozen in liquid nitrogen and stored at −80°C until further analysis.

### Measurement of E and NE Levels

Blood samples from the nude mice were collected and centrifuged for 15 min at 4,000 g and 4°C. The resultant plasma was kept at −80°C until analysis. E and NE levels were measured using an enzyme immunoassay kit from DRG International (Austin, TX, USA) according to the manufacturer's instructions, as previously described by Guo et al. [33]. Absorbance was measured with the microplate reader at 450 nm. The plasma concentrations of E and NE were expressed as nanograms per milliliter (ng/ml) of plasma.

### Western blotting

The cells were treated as indicated, and cell lysates were prepared with a cell lysis buffer (20 mM Tris-Cl (pH 7.5), 150 mM NaCl, 1 mM EDTA, 1 mM EGTA, 1% Triton X-100, 2.5 mM sodium pyrophosphate, 1 mM β–glycerolphosphate, 1 mM Na_3_VO_4_, and 1 µg/ml leupeptin) with 1 mM phenylmethylsulfonyl fluoride added before use. Equal amounts of protein were resolved by 10% SDS-PAGE, transferred to a nitrocellulose membrane from Amersham Corporation (Pittsburgh, PA,USA), blocked in 5% skimmed milk in TBS-T (0.1% Tween 20 in Tris-buffered saline), probed with primary antibodies overnight at 4°C and incubated for 1 hour with secondary peroxidase-conjugated antibodies from Jackson ImmunoResearch(West Grove, PA, USA)(1∶5000) at room temperature. The membranes were developed with an enhanced chemiluminescence system from Pierce Biotechnology Inc. (Rockford, IL, USA). GAPDH was probed as an internal control.

### Reverse Transcriptase-Polymerase Chain Reaction (RT-PCR)

Total RNA was isolated from CRC cells using the TRIzol Reagent according to the manufacturer's instructions from Invitrogen, Life Technologies (Grand Island, NY, USA). First-strand cDNAs were synthesized by M-MLV reverse transcriptase from Fermentas, Thermo Fisher Scientific, Inc. (Waltham, MA, USA) and subjected to PCR by specific primers for the human β1-AR (ADRB1) gene (forward, 5′- CTCCTTCTTCTGCGAGCTGTGGA -3′; reverse, 5′- ATGAGGATGGGCAGGAAGGACA -3′), β2-AR (ADRB2) gene (forward, 5′-CATGTCTCTCATCGTCCTGGCCA-3′; reverse, 5′- CACGATGGAAGAGGCAATGGCA-3′) and β3-AR (ADRB3) gene (forward, 5′- GCCCAATACCGCCAACAC-3′; reverse, 5′-GCCAGCGAAGTCACGAACAC-3′). The PCR conditions for β1-AR included an initial incubation at 94°C for 12 min, followed by 38 cycles of 94°C for 1 min, 60°C for 3 min, and 72°C for 1 min. The PCR conditions for β2-AR included an initial incubation at 94°C for 12 min; 38 cycles of 94°C for 1 min, 60°C for 2 min, and 72°C for 3.5 min; and a final incubation at 72°C for 5 min. The PCR conditions for β3-AR included an initial incubation at 95°C for 2 min; 34 cycles of 95°C for 30 seconds, 56°C for 30 seconds, and 72°C for 1 min; and a final incubation at 72°C for 5 min. The PCR products were visualized on a 1.2% agarose gel stained with ethidium bromide.

### Immunohistochemical staining

After euthanizing the nude mice at the end of the experiments, tumor tissues were harvested, fixed in 10% neutral-buffered formalin, embedded in paraffin and sectioned at 5 µm. For immunohistochemical staining, the sections were deparaffinized and rehydrated, and then, antigen retrieval was performed by boiling the samples in a citrate buffer for 15 min at 92 °C–98 °C. The sections were immersed in distillated water containing 3% hydrogen peroxide for 30 min to block endogenous peroxidase activity. Nonspecific staining was eliminated with normal goat serum from Invitrogen, Life Technologies (Grand Island, NY, USA). The sections were incubated with the primary antibodies against PCNA, Ki-67, or phospho-ERK1/2 at 37 °C for 2 hours and then washed three times for 20 min each with PBS containing Tween-20. The sections were incubated with biotin-labeled secondary antibodies, followed by incubation with an avidin-biotin complex (ABC) from DAKO (Denmark) for 30 min at 37 °C. The nuclei were counterstained with hematoxylin. The remaining immunohistochemical staining procedures were performed following the manufacturer's instructions. The slides were quantified using the Image-Pro Plus software from Media Cybernetics (Rockville, MD, USA), as previously described by Augsten et al. [34].

### Statistical analysis

If normally distributed, continuous variables between two groups were compared with Student's t test, whereas comparisons between three or more groups were performed with one-way analysis of variance (ANOVA) followed by Tukey's test. Otherwise, nonparametric tests, such as Tukey's test, if appropriate, were used to compare the differences. P values less than 0.05 were considered to be statistically significant.

### Ethics statement

All animal experiments were performed in accordance with the Guide for the Care and Use of Laboratory Animals (the "NIH Guide"). The protocols for the use of animals were approved by the Department of Laboratory Animal Sciences, SJUSM (permit numbers SYXK (Hu) 2008 0050).

## Results

### Effect of CRS on CRC growth in vivo

**Figure 1:**
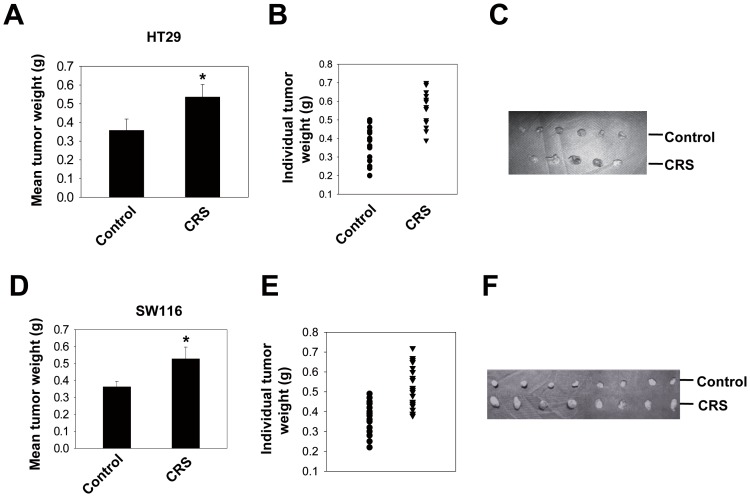
Effect of CRS on CRC growth in vivo. Mice were injected subcutaneously (s.c.) with CRC HT29 cells (2×106) in the dorsal flank 7 d after starting the stress treatment. Daily CRS treatment was continued for an additional 21 d. Mean tumor weight (A), individual tumor weight (B) and representative tumor images (C) from the CRS group and the no-stress control group are shown (* P = 0.028; n = 16–17; symbols represent individual mice; for each treatment group, the mean ± SD of three independent experiments is shown). The same experimental protocol was applied to mice inoculated with SW116 cells. Mean tumor weight (D), individual tumor weight (E) and representative tumor images (F) are shown (* P = 0.021; n = 20; symbols represent individual mice; for each treatment group, the mean ± SD of three independent experiments is shown).

We first examined the effect of CRS on tumor growth in vivo. HT29 or SW116 cells were inoculated into nude mice 7 d after the initiation of CRS, as described in the Materials and Methods section. Twenty-one days after tumor cell inoculation, CRS treatment significantly increased tumor weight (Fig. 1A, B, D and E) and tumor size (Fig. 1C and F) in both HT29-inoculated and SW116-inoculated nude mice. To investigate whether the proliferation of tumor cells was increased by CRS, we quantified the number of PCNA-positive cells. PCNA immunostaining of tumor tissues from both the control and CRS groups revealed that CRS promoted HT29 cell growth in nude mice (Fig. S1B). To further confirm this result, we also performed immunostaining of Ki-67 and found results comparable to those of the PCNA experiment (Fig. S2B).

### Effect of CRS on E and NE levels and on heart, liver, spleen and body weights

**Figure 2:**
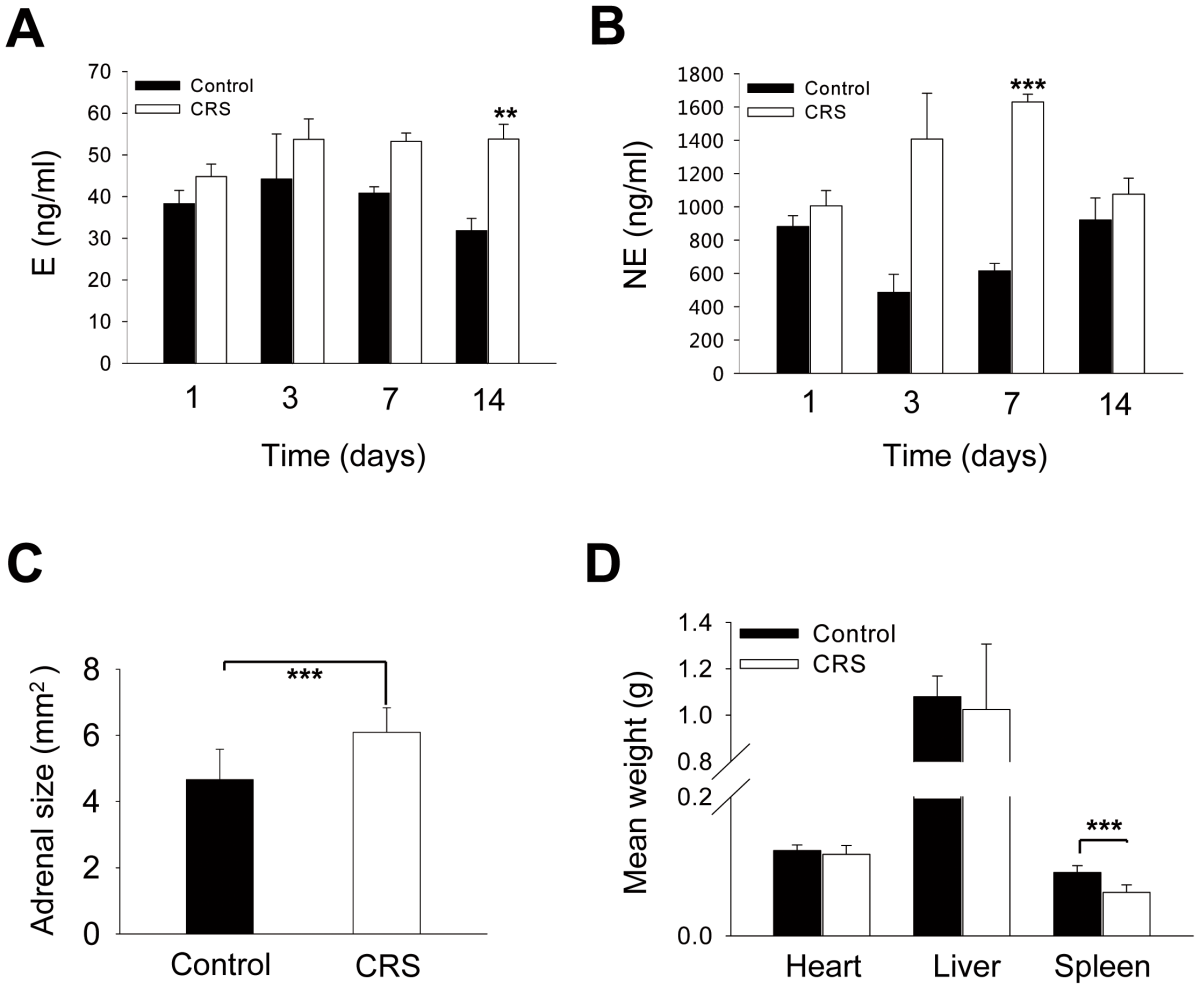
Effect of CRS on E and NE levels and on heart, liver, spleen weights and adrenal size. After 1 week of habituation to the vivarium setting, animals received 1, 3, 7, or 14 d of CRS for 6 h daily, after which E (A) and NE (B) levels were assayed by ELISA, as described in Materials and Methods section (** P = 0.003, *** P<0.001, significantly different from the no-stress control group; n = 3; for each time point, the mean ± SD is shown). Adrenal size (C) (mm^2^, perpendicular diameter of individual adrenal gland) and the heart, liver, spleen weights (D) from the no-stress control group and the CRS group (*** P<0.001, significantly different from the no-stress control group; n = 16–17, for each group, the mean ± SD is shown).

To confirm that our CRS protocol did produce stress to the mice, we determined the plasma concentrations of the stress hormones E and NE in nude mice subjected to CRS for 1, 3, 7, or 14 days after tumor cell inoculation. We found that CRS increased both E and NE concentrations but did so over different time courses (Fig. 2A and B). At the end of the experiments, however, there was no significant difference in body weight between the CRS mice and the no-stress control mice (Fig. S3), indicating that the physical health condition of the animals was not different between the groups.

To assess the effects of CRS on sympatho-adrenal-medullary activity, we measured the size of both adrenal glands. In the animals inoculated with HT29 cells, the left adrenal glands of the CRS group were significantly larger than those of the control group (6.103±0.745 mm^2^ vs. 4.661±0.92 mm^2^, P<0.001) (Fig. 2C). Similar results were also found for the right adrenal gland. The weight of the spleen was remarkably decreased in the CRS group, whereas CRS had no obvious effect on the heart and liver weights (Fig. 2D).

### Effect of stress hormones on CRC growth in vivo

We have shown that CRS could promote CRC cell growth in vivo and stimulate the release of stress hormones. We then investigated if increased levels of stress hormones was responsible for the promotion of CRC cell growth in vivo. A previous study showed that chronic stress establishes favorable conditions for promoting tumor growth by SNS activation [22]. Because E was more persistently increased by CRS in our study (Fig. 2A), we investigated the effect of E on CRC growth in vivo. Different doses of E were applied using microosmotic pumps to examine the effect of E on tumor cell growth. Considering the prevalent use of HT29 cells in xenograft experiments and the similar responses to CRS between HT29 and SW116 cells (Fig. 1), we examined the effect of E on HT29 cell proliferation in nude mice.

**Figure 3:**
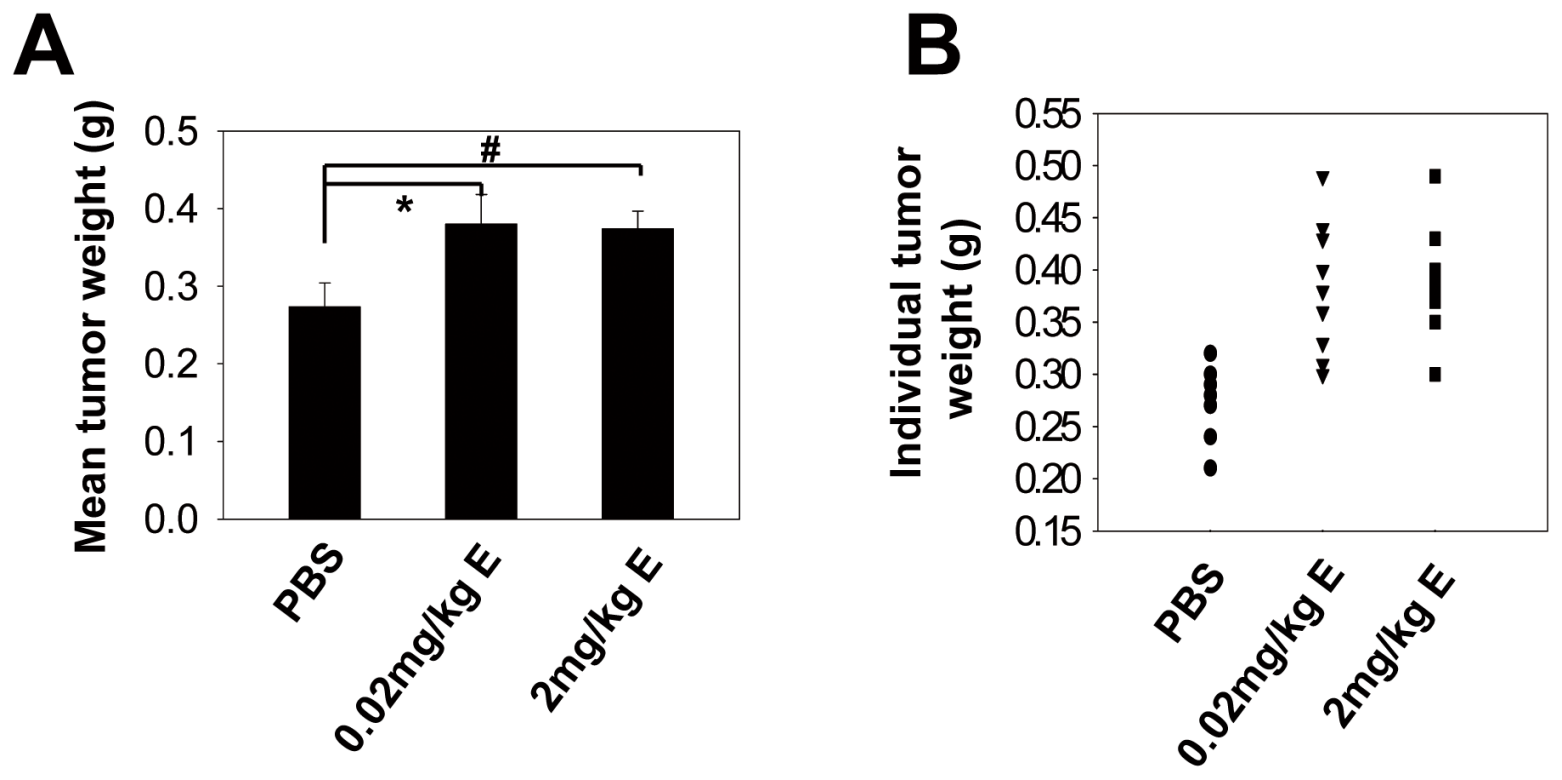
Adrenergic activity in response to CRS promotes in vivo CRC growth. (A) HT29-inoculated mice were treated with PBS, 0.02 mg/kg E or 2 mg/kg E. The mean tumor weight of each treatment group was measured (* P = 0.034, ^#^ P = 0.043; n = 5–6; for each group, the mean ± SD of one of two experiments is shown). (B) Individual tumor weights of the three different treatment groups are also shown (n = 11; symbols represent individual mice).

Animals were treated with (1) control PBS, (2) E at 0.02 mg/kg, and (3) E at 2 mg/kg. Treatment began 4 d after the injection of HT29 cells. We found that the lower dose (0.02 mg/kg) was able to saturate the in vivo tumor-promoting effect of E (Fig. 3A and B), indicating that the level of E released in our CRS experiment was sufficiently high to induce tumor growth. Accordingly, immunohistochemical analyses of PCNA (Fig. S1A) and Ki-67 (Fig. S2A) revealed that E promoted HT29 cell proliferation in vivo. Together, these data suggest that the stress hormones induced by our CRS protocol could enhance CRC cell growth in vivo.

### Effect of stress hormones on the proliferation of CRC cells in vitro

**Figure 4:**
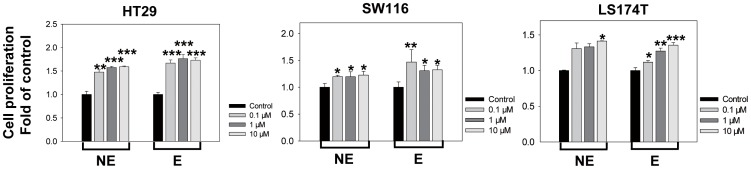
Effects of E or NE on CRC cells Proliferation. CRC HT29, SW116 and LS174T cell lines were treated with different concentrations of E or NE, as indicated for 24 h, respectively, cell proliferation was measured by BrdU incorporation assay, as described in the Materials and Methods section. Data are expressed as mean ± SD of one representative of at least three experiments. HT29, (0.1 µM NE, ** P = 0.0011; *** P<0.001, significantly different from the control group); SW116, (0.1 µM NE, * P = 0.034; 1 µM NE, * P = 0.034; 10 µM NE, * P = 0.017; 0.1 µM E, ** P = 0.0037; 1 µM E, * P = 0.030; 10 µM E, * P = 0.023, significantly different from the control group); LS174T, (10 µM NE, * P = 0.025; 0.1 µM E, * P = 0.023; 1 µM E, ** P = 0.005; 10 µM E, *** P<0.001, significantly different from the control group).

To validate the tumor-promoting effect of the stress hormones, we further tested the effect of hormones on cell proliferation in vitro. HT29, SW116 and LS174T cells were treated with various concentrations of the stress hormones E, NE or corticosterone (glucocorticoid in the murine), and the cell proliferation of all three cell lines was examined using the BrdU incorporation assay. We found that both E and NE markedly increased the proliferation of all three CRC cell lines in a dose-dependent manner (Fig. 4). However, no significant differences in proliferation were found when the tumor cells were cultured in the presence of corticosterone (CORT) at increasing concentrations (Fig. S4), indicating that E and NE were the stress hormones specifically associated with CRC growth. To further validate the growth-promoting effect of the stress hormones, we evaluated the impact of E and NE on the viability of all three CRC cell lines. The percentage of viable cells, as determined by the CCK-8 assay, was significantly increased in E- and NE-treated CRC cells in a dose-dependent manner when compared with the untreated control (Fig. S5).

### β-AR antagonists block stress hormone-induced CRC cell proliferation in vitro and in vivo

**Figure 5:**
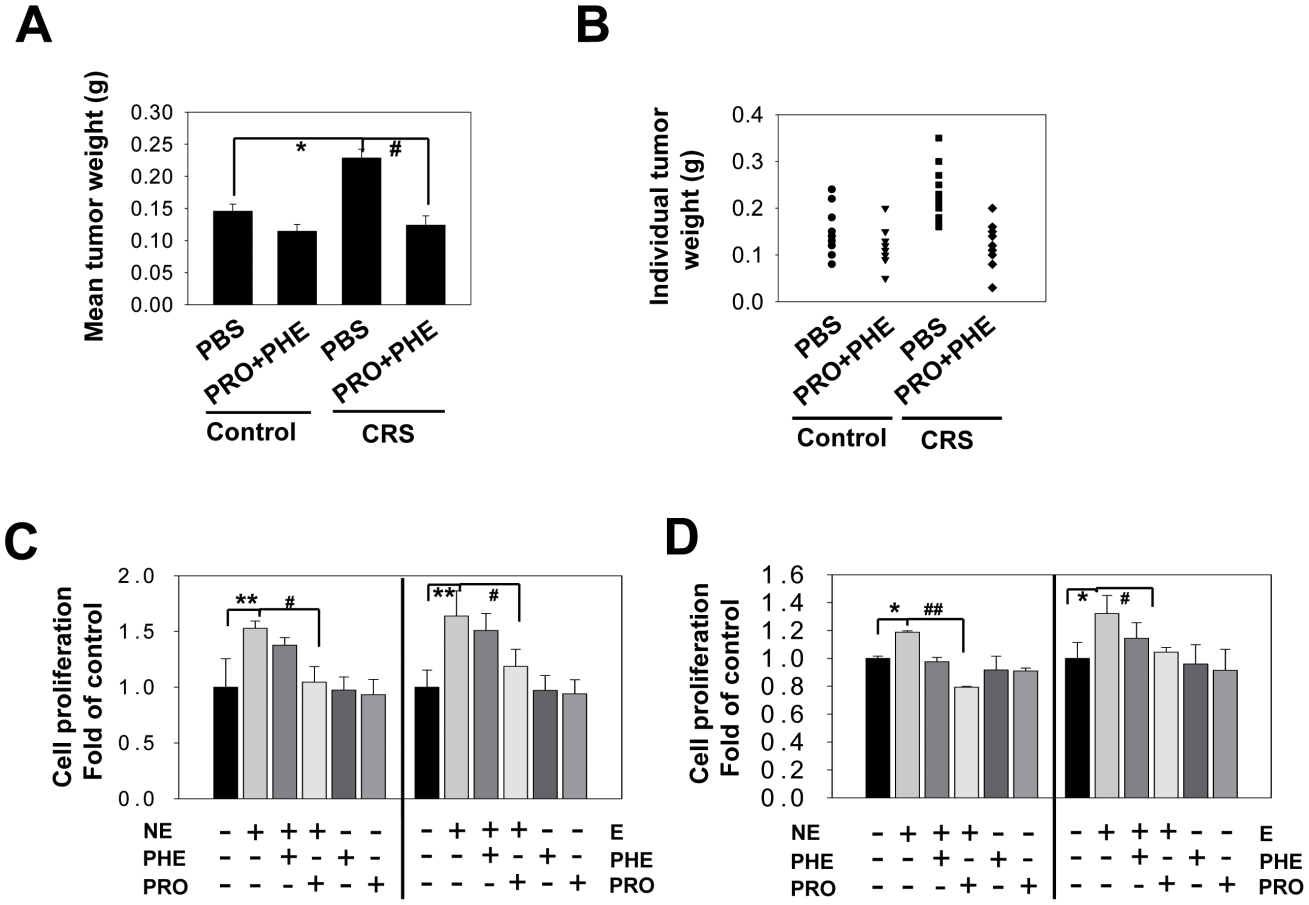
E or NE-induced CRC cell proliferation is both α- and β-AR dependent. HT29-inoculated mice were treated with PBS or a combination of the α-AR antagonist PHE (2 mg/kg) and β-AR antagonist PRO (2 mg/kg) under CRS or no stress. The mean tumor weight (A) of each treatment group was measured (* P = 0.038, ^#^ P = 0.015; n = 11–12; for each group, the mean ± SD of two independent experiments is shown) and individual tumor weights (B) of the four different treatment groups are also shown (n = 11–12; symbols represent individual mice). HT29 (C) and SW116 cells (D) were pretreated with or without the α-AR antagonist PHE (50 µM) or β-AR antagonist PRO (50 µM) for 45 min before incubation with NE (10 µM) or E (10 µM). After 24 h, cell proliferation was measured by the BrdU incorporation assay, as described in the Materials and Methods section. The data are expressed as the mean ± SD of triplicate or quadruplicate samples per treatment group from at least three independent experiments with similar results. (A) NE, (** P = 0.007, ^#^ P = 0.022); E, (** P = 0.004, ^#^ P = 0.043); (B) NE, (* P = 0.034, ^##^ P = 0.009); E, (* P = 0.013, ^#^ P = 0.032).

E and NE, the major hormones released under stress conditions, can bind to specific α- or β-AR [35], [36], [37]. We investigated whether CRS-induced CRC growth is dependent on AR. To do so, we examined whether α- and β-AR antagonists can block the stimulatory effects of CRS on CRC growth in nude mice. We found that the nonselective α-AR antagonist (PHE) and β-AR antagonist (PRO) blocked CRS-induced tumor growth in vivo (Fig. 5A and B). Immunohistochemical staining for PCNA (Fig. S1B) and Ki-67 (Fig. S2B) further supported the antagonistic effect of PHE and PRO on CRS-induced tumor cell proliferation, indicating that CRS acted in an AR-dependent manner to induce CRC growth in vivo.

Because stress hormones can activate multiple β-AR subtypes, we investigated which β-AR subtype was involved in promoting tumor cell proliferation. We first examined the β1-AR, β2-AR and β3-AR mRNA and protein levels in the CRC cell lines. We were able to detect β1- and β2-AR mRNA expression in all CRC cell lines, while β3-AR was not detected in any cell line (Fig. S6A). The protein expression of β1-AR and β2-AR was also confirmed by Western blot in these cell lines (Fig. S6B).

To investigate the involvement of specific subtypes of AR in the stimulatory effect of the stress hormones on CRC cell proliferation in vitro, HT29 and SW116 cells were treated with E or NE in the presence of either PHE or PRO. We found that the stress hormones significantly induced cell proliferation and that the PRO treatment significantly reversed this effect in both HT29 (Fig. 5C) and SW116 cells (Fig. 5D). To address whether β-AR stimulation could promote tumor cell proliferation, HT29 and SW116 cells were treated with the β-AR agonist ISO at increasing concentrations. We found that the growth-promoting effect of ISO was saturated at 1 µM (Fig. S7A); thus, we applied the same dose in the subsequent experiments. We also found that β-AR blockade could reverse the effect of ISO (Fig. S7B).

**Figure 6:**
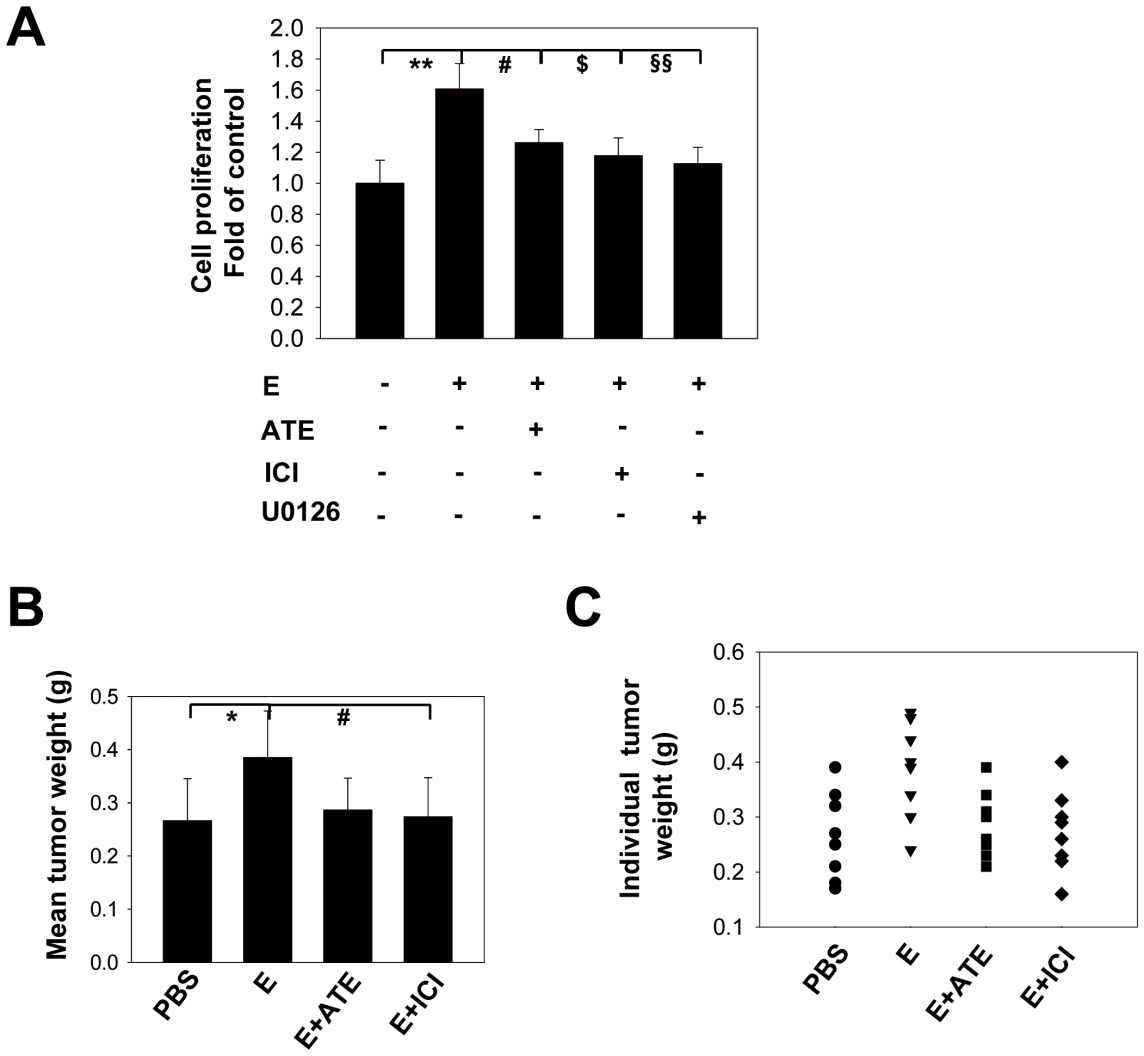
β-AR-mediated CRC cell proliferation and growth in mice. (A) HT29 cells were pretreated with the β1-AR antagonist ATE (50 µM) or β2-AR antagonist ICI (50 µM) for 45 min before incubation with E (10 µM). After 24 h, cell proliferation was measured by the BrdU incorporation assay, as described in the Materials and Methods section. The data are expressed as the mean ± SD of triplicate or quadruplicate samples per treatment group from at least three independent experiments with similar results. (** P = 0.001, ^#^ P = 0.045, ^$^ P = 0.013, ^§§^P = 0.006) (B) HT29-inoculated mice were treated with PBS (used as control), E (0.02 mg/kg), E plus ATE (5 mg/kg), or E plus ICI (5 mg/kg). The mean tumor weight of each group was measured at the end of the experiment (* P = 0.02, ^#^ P = 0.032; n = 8; means ± SDs are shown). (C) Individual tumor weights of each group are also shown (n = 8; symbols represent individual mice).

In cell proliferation experiments, both the selective β1-AR antagonist ATE and β2-AR antagonist ICI significantly blocked E-induced cell proliferation (Fig. 6A), indicating the involvement of both β-AR subtypes in promoting tumor cell proliferation. These results were further validated by the in vivo experiments (Fig. 6B and C) and the immunostaining for PCNA (Fig. S1C) and Ki-67 (Fig. S2C).

### ERK1/2-specific inhibitor U0126 abolished the stimulatory effect of β-AR on cell proliferation

To further understand the molecular mechanisms involved in β-AR-mediated tumor cell proliferation, we examined the role of ERK1/2 by treating cells with a specific ERK1/2 inhibitor U0126. Compared with the E-alone group, E+U0126 showed a significantly lower cell proliferation index, indicating a critical role of ERK1/2 in promoting CRC tumor cell growth (Fig. 6A). To demonstrate the effectiveness of U0126 in inhibiting β-AR-mediated cell proliferation, we directly stimulated β-AR with ISO and found that U0126 significantly reduced the effect of ISO (Fig. S7C).

### β-AR antagonists abrogated E-induced hyperphosphorylation of ERK1/2

**Figure 7:**
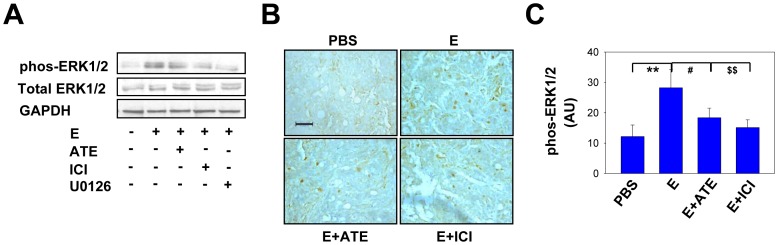
E-induced hyperphosphorylation of ERK1/2 can be abolished by β-AR antagonists. (A) HT29 cells were treated with E (10 µM) in the absence or presence of ATE (50 µM), ICI (50 µM) or U0126 (20 µM) for 45 min as indicated, and the cell lysates were homogenized for the immuno-detection of phos-ERK1/2 by Western blot. (B) HT29 tumor samples from the PBS (used as control), E (0.02 mg/kg), E plus ATE (5 mg/kg), and E plus ICI (5 mg/kg) treatments were immunohistochemically stained for phos-ERK1/2. The representative tumor sections are illustrated. (C) The quantified values represent the average immunostaining intensities of phos-ERK1/2 from at least five random fields under 400× magnification (scale bar, 50 µm). Under microscopy, a brown color indicates positive immunostaining (** P = 0.002, ^#^ P = 0.029, ^$$^ P = 0.005; mean ± SD are shown).

Previous studies have shown that ERK1/2 phosphorylation and the subsequent activation of downstream pathways might be involved in stimulating CRC cell proliferation [38], [39], [40], [41], [42]. We therefore determined whether the phosphorylation level of ERK1/2 in HT29 cells was increased upon E treatment and found that E significantly increased ERK1/2 phosphorylation, which was reversible by β1-AR, β2-AR and ERK1/2 antagonists. The total ERK1/2 expression was unaffected by any drug treatment in this study (Fig. 7A). The immunohistochemical staining results revealed that phosphorylation of ERK1/2 in the tumor tissues from the HT29-inoculated mice treated with E was significantly enhanced in comparison with the PBS control group (Fig. 7B and C). We also found that both the β1-AR selective antagonist ATE and the β2-AR selective antagonist ICI significantly decreased the effect of E on ERK1/2 phosphorylation (Fig. 7B and C).

## Discussion

Stressful experiences may be caused by physical stressors (such as pathogens and toxins) and psychological stressors (such as major life events, trauma, abuse, or factors related to the living environment). Short-term stress response is proposed to be beneficial for the adaptation of the organism, while long-term exposure to stress ultimately causes harm to the individual [43]. Chronic stress in both animals and humans has been shown to decrease cellular immune parameters [44], [45], [46]. Stress has been suggested to play a central role in the incidence and progression of cancers. Available results show that exposure to chronic stress has specific effects on the immune system and concomitantly influences tumor growth and cancer cell functions [4], [22], [46], [47].

A wide variety of stresses have been used to study the effects of stress on tumor biology. Exposure to stress conditions such as surgery has been shown to promote tumor growth [48] and augment tumor development and metastasis in animal models [21], [49]. Recent evidence confirmed that both social isolation [22], [50] and CRS [22], [51], [52] promoted tumor growth and progression. Furthermore, the CRS animal model has become the preferred choice for studying the effect of stress on tumor growth and progression. Stressors activate major neural pathways, the sympathetic nervous system (SNS) and the hypothalamic-pituitary-adrenal (HPA) axis to release stress hormones [3], [4], [5]. Stress hormones might directly regulate the growth and metastatic potential of tumor cells, and this effect might be independent of the immune system [22]. In the present study, we investigated whether CRS-induced elevation of E and/or NE may increase CRC growth in nude mice.

Our results demonstrated that CRS significantly promoted CRC tumor growth in an experimental mouse model. The representative stress hormone E, when administered at 0.02 mg/kg (low dose) and 2 mg/kg (high dose; ∼40% of its LD_50_, [53]), significantly enhanced HT29 cell growth in vivo when compared to the PBS treatment. In addition, a combination of α- and β-AR antagonists blocked the stimulatory effects of CRS on HT29 cell growth in mice, indicating the involvement of AR-dependent pathways. The in vivo results were consistent with our in vitro data showing that E/NE enhanced human CRC cell proliferation and viability through AR-dependent pathways. Together, our results provided strong support for the role of stress hormones in promoting CRC cell growth upon CRS stimulation.

The doses of E and NE used in the in vitro experiments reflect their physiological levels in tumors. Circulating plasma levels of catecholamines range from 10 to 1,000 pM in a normal individual and may reach 100 nM under conditions of stress [6]. Moreover, studies suggest that the concentrations may reach as high as 10 µM in a tumor microenvironment, such as within the parenchyma of the ovary [54], [55]. In this study, we examined the effects of four different concentrations (0, 0.1 µM, 1 µM and 10 µM) to cover the entire range of possible physiological concentrations of stress hormones. Therefore, our CRS model was sufficient to test the effect of physiological stress on tumor growth.

Our CRS protocol (6 h stress) resulted in elevated E and NE levels in the plasma [56], [57], reduced spleen weight [20] and enlarged adrenal glands [20], [22] compared with the no-stress control. All of these parameters confirmed the effectiveness and reliability of our CRS mouse model. In the present study, we did not observe any significant tumor-promoting effect when using a CRS protocol with a 3 h daily stress exposure (data not shown). Similarly, Wong et al. [57] reported that restraint stress alone (1 h daily for 33 days) did not significantly promote CRC tumor growth in a similar xenograft model, while a combined exposure with cigarette smoke remarkably promoted tumor growth. Indeed, different levels of stress exposure could have different effects on cancer development [58]. Moreover, another recent study showed no significant correlation between chronic restraint stress in mice and the incidence and severity of oral squamous cell carcinoma (OSSC) [29]. Such differences in tumor response to stress might be due to the involvement of different immune components, the types of tumors investigated, the species of animals used, and/or the levels of stress produced. Thus, a better understanding of the molecular mechanisms that occur under different conditions will help to reconcile the variable relationships between stress and tumor development.

In addition to the catecholamines, glucocorticoid is also a type of classic stress hormone. In this experiment, we found that corticosterone did not markedly increase the proliferation of three CRC cell lines at any of the tested dose, suggesting that the HPA/glucocorticoid axis may not be implicated in CRS-mediated tumor growth. However, it should be noted that these findings do not rule out the possibility that other neuroendocrine signaling pathways might modulate tumor development under different circumstances. For example, stress can affect the hypothalamic-pituitary-thyroid axis [59], the inhibitory effects of dopamine on VEGF activity [53] and tumor growth [53], [60], [61].

Considering the critical role of stress in regulating tumor growth and the fact that stress hormones and their antagonists could be quickly metabolized, we used microosmotic pumps instead of repeated injection to administer the stress hormones and antagonists to reduce the stress response as much as possible during manipulations of the animals. Indeed, excessive surgical stress has been shown to augment cancer growth and metastasis [48]. Therefore, a single application of the pumps in this study allowed us to minimize the potential side-effects on the experimental system due to surgery.

Our results suggested a prominent role of β-AR in cell proliferation, consistent with the reported growth inhibitory effects of β-AR blockade [26], [52], [62], [63], [64], [65], [66], [67], [68], [69]. We also observed that the positive effect of E on CRC cell proliferation was blocked by both β1- and β2-AR antagonists, which is consistent with certain previous findings [27], [40], [70], [71].

In our attempt to identify the downstream signaling pathways involved in AR-mediated tumor growth, we found that β1- and β2-AR antagonists blocked E-induced phosphorylation of ERK1/2. Indeed, it has been reported that activation of β-AR promoted cell proliferation, which was accompanied by ERK/MAPK pathway activation [72], [73], [74], [75]. Previous research also indicated an especially critical role of β2-AR in the activation of the ERK/MAPK signaling pathway [26], [70]. Therefore, we conclude that β-AR-mediated ERK1/2 activation could be one mechanism mediating CRS-induced cancer cell growth in vivo. Our results also suggest that β-AR blockade may be an effective preventive and therapeutic approach for patients with stress-related CRC.

## Supporting Information

Figure S1 **Immunohistochemical staining for PCNA.** Immunohistochemical staining for PCNA was performed on (A) HT29 tumor samples from the PBS, 0.02 mg/kg E and 2 mg/kg E treatment groups; (B) HT29 tumor samples from the mice groups treated with PBS or PHE (α-AR antagonist) plus PRO (β-AR antagonist) under CRS or no stress; and (C) HT29 tumor samples from the mice groups treated with PBS, E (0.02 mg/kg), E plus ATE (β1-AR antagonist, 5 mg/kg), or E plus ICI (β2-AR antagonist, 5 mg/kg). Representative tumor sections (left upper panel) and high magnification images of selective portions (left lower panel) from each group are shown. The quantitative data in the graph correspond to the left-side images (right panel). Under microscopy, a dark brown color indicates strong positive immunostaining. Quantified values represent the average immunostaining intensities from at least five random fields of each slide from each tumor tissue, and three to five random tumor tissues from each treatment group are included (magnification: ×400). (Scale bar 50 µm). The data were mean ± SD. * P<0.05, significantly different from the PBS control group; ^$$^ P<0.01 significantly different from the CRS group and ^#^ P<0.05, ^##^ P<0.01significantly different from the E-treated group.
(TIF)

Figure S2 **Immunohistochemical staining for Ki-67.** Immunohistochemical staining for ki-67 was performed on (A) HT29 tumor samples from PBS, 0.02 mg/kg and 2 mg/kg E treatment groups, (B) HT29 tumor samples from PBS, combination of α-AR antagonist PHE with β-AR antagonist PRO treatments under no-stress or CRS, respectively, and (C) HT29 tumor samples from PBS (used as control), E (0.02 mg/kg), E combined with β1-AR antagonist ATE (5 mg/kg), E combined with β2-AR antagonist ICI (5 mg/kg) treatments were subjected to immunohistochemical staining for Ki-67. Illustrated from each group were representative tumor sections (left panel). The quantitative data in the graph correspond to the left images were shown (right panel). Under microscopy, dark brown color indicates strong positive immunostaining. Quantified values shown were the average immunostaining intensity counted in at least five random fields of each slide from each tumor tissue, and three to five random tumor tissues from each treatment group were included, magnification (×400). (Scale bar 50 µm). The data were mean ± SD. * P<0.05, ** P<0.01, significantly different from the no-stress control group; ^$^ P<0.05 significantly different from the CRS group and ^#^ P<0.05 significantly different from the E-treated group.
(TIF)

Figure S3 **Effect of CRS on body weight of mice.** After 1 week of habituation to the vivarium setting, mice subjected to the CRS group or no-stress control group were inoculated subcutaneously (s. c.) with CRC cells into the dorsal flank. Daily CRS was continued for an additional 21 d. Mice (n = 16–17 per group) were weighed and recorded every three days, as indicated. No obvious difference was found in body weight of mice between the CRS group and the no-stress control group.
(TIF)

Figure S4 **Effects of corticosterone on CRC cells proliferation.** CRC HT29, SW116 and LS174T cell lines were treated with different concentrations of corticosterone, as indicated for 24 h, respectively, cell proliferation was measured by BrdU incorporation assay, as described in the materials and methods section. No significant difference was found in any cell line. Data are expressed as mean ± SD of one representative of at least three experiments.
(TIF)

Figure S5 **Effects of E or NE on CRC cells viability.** The CRC HT29, SW116 and LS174T cell lines were seeded in 96-well plates and cultured in the presence of different concentrations of E or NE for 24 h, as indicated. Cell viability was measured by CCK-8 assay, as described in the Materials and Methods section. Both E and NE significantly promoted all three CRC cell lines survival in a dose-dependent manner. The results are expressed as mean ± SD of one representative of three independent experiments. * P<0.05, ** P<0.01, *** P<0.001 significantly different from the control group.
(TIF)

Figure S6 **Expression of β1- and β2-AR in CRC cell lines.** (A) RT-PCR was performed to determined β1-, β2-AR and β3-AR mRNA levels in CRC HT29, SW116 and LS174T cells. Representative RT-PCR assay were shown. (B) Lysates from CRC HT29, SW116 and LS174T cell lines using specific monoclonal antibody probed for β1-AR and polyclonal antibody probed for β2-AR. Both β1- and β2-AR protein expressed in CRC HT29, SW116 and LS174T cell lines by western blot analysis.
(TIF)

Figure S7 **Involvement of ERK1/2 in the β-AR-mediated CRC cells proliferation.** (A) HT29 (left panel) and SW116 cells (right panel) were treated with different concentrations of β-AR agonist ISO, as indicated for 24 h, respectively. Result of BrdU incorporation assays showed ISO remarkably induced both CRC cells proliferation at dose of 1 µM. Data are expressed as mean ± SD of one representative of three experiments. * P<0.05, ** P<0.01 significantly different from the control group. (B) HT29 (left panel) and SW116 cells (middle panel) were pretreated with β-AR antagonist PRO, β1-AR antagonist ATE (50 µM) or β2-AR antagonist ICI (50 µM) for 45 min before incubation with β-AR agonist ISO (1 µM), respectively. After 24 h, cell proliferation was measured by BrdU incorporation assay, as described in the materials and methods section. Data were expressed as mean ± SD of triplicate or quadruplicate samples per treatment group in at least three independent experiments with similar results. * P<0.05 significantly different from the control group and ^#^ P<0.05, ^##^ P<0.01, significantly different from the ISO-treated group, (C)HT29 cells were pretreated with or without ERK1/2 specific inhibitor U0126 (20 µM) for 45 min before incubation with ISO (1 µM). Results showed that ISO-induced cell proliferation was remarkably blocked by ERK1/2 specific inhibitor U0126. Data were expressed as mean ± SD of triplicate or quadruplicate samples per treatment group in at least three independent experiments with similar results. * P<0.05, significantly different from the control group and ^#^ P<0.05, significantly different from the ISO-treated group.
(TIF)

## References

[1] Tanaka T (2009) Colorectal carcinogenesis: Review of human and experimental animal studies. J Carcinog 8: 5. 19332896 10.4103/1477-3163.49014 PMC2678864

[2] Courtney JG, Longnecker MP, Peters RK (1996) Psychosocial aspects of work and the risk of colon cancer. Epidemiology 7: 175–181. 8834558 10.1097/00001648-199603000-00012

[3] Glaser R, Kiecolt-Glaser JK (2005) Stress-induced immune dysfunction: implications for health. Nat Rev Immunol 5: 243–251. 15738954 10.1038/nri1571

[4] Antoni MH, Lutgendorf SK, Cole SW, Dhabhar FS, Sephton SE, et al (2006) The influence of bio-behavioural factors on tumour biology: pathways and mechanisms. Nat Rev Cancer 6: 240–248. 16498446 10.1038/nrc1820 PMC3146042

[5] Thaker PH, Lutgendorf SK, Sood AK (2007) The neuroendocrine impact of chronic stress on cancer. Cell Cycle 6: 430–433. 17312398 10.4161/cc.6.4.3829

[6] Schmidt C, Kraft K (1996) Beta-endorphin and catecholamine concentrations during chronic and acute stress in intensive care patients. Eur J Med Res 1: 528–532. 9438155

[7] Rupp H, Dhalla KS, Dhalla NS (1994) Mechanisms of cardiac cell damage due to catecholamines: significance of drugs regulating central sympathetic outflow. J Cardiovasc Pharmacol 24 Suppl 1S16–24. 10.1097/00005344-199424001-00004 7533222

[8] Vandewalle B, Revillion F, Lefebvre J (1990) Functional beta-adrenergic receptors in breast cancer cells. J Cancer Res Clin Oncol 116: 303–306. 2164516 10.1007/BF01612908 PMC12201344

[9] Marchetti B, Spinola PG, Pelletier G, Labrie F (1991) A potential role for catecholamines in the development and progression of carcinogen-induced mammary tumors: hormonal control of beta-adrenergic receptors and correlation with tumor growth. J Steroid Biochem Mol Biol 38: 307–320. 1848992 10.1016/0960-0760(91)90102-b

[10] Badino GR, Novelli A, Girardi C, Di Carlo F (1996) Evidence for functional beta-adrenoceptor subtypes in CG-5 breast cancer cell. Pharmacol Res 33: 255–260. 8938018 10.1006/phrs.1996.0036

[11] Enserink JM, Price LS, Methi T, Mahic M, Sonnenberg A, et al (2004) The cAMP-Epac-Rap1 pathway regulates cell spreading and cell adhesion to laminin-5 through the alpha3beta1 integrin but not the alpha6beta4 integrin. J Biol Chem 279: 44889–44896. 15302884 10.1074/jbc.M404599200

[12] Masur K, Niggemann B, Zanker KS, Entschladen F (2001) Norepinephrine-induced migration of SW 480 colon carcinoma cells is inhibited by beta-blockers. Cancer Res 61: 2866–2869. 11306460

[13] Lang K, Drell TLt, Lindecke A, Niggemann B, Kaltschmidt C, et al (2004) Induction of a metastatogenic tumor cell type by neurotransmitters and its pharmacological inhibition by established drugs. Int J Cancer 112: 231–238. 15352035 10.1002/ijc.20410

[14] Sood AK, Bhatty R, Kamat AA, Landen CN, Han L, et al (2006) Stress hormone-mediated invasion of ovarian cancer cells. Clin Cancer Res 12: 369–375. 16428474 10.1158/1078-0432.CCR-05-1698 PMC3141061

[15] Reiche EM, Nunes SO, Morimoto HK (2004) Stress, depression, the immune system, and cancer. Lancet Oncol 5: 617–625. 15465465 10.1016/S1470-2045(04)01597-9

[16] Kojima M, Wakai K, Tokudome S, Tamakoshi K, Toyoshima H, et al (2005) Perceived psychologic stress and colorectal cancer mortality: findings from the Japan Collaborative Cohort Study. Psychosom Med 67: 72–77. 15673627 10.1097/01.psy.0000151742.43774.6d

[17] Allison PJ, Guichard C, Fung K, Gilain L (2003) Dispositional optimism predicts survival status 1 year after diagnosis in head and neck cancer patients. J Clin Oncol 21: 543–548. 12560447 10.1200/JCO.2003.10.092

[18] Reynolds P, Kaplan GA (1990) Social connections and risk for cancer: prospective evidence from the Alameda County Study. Behav Med 16: 101–110. 2224168 10.1080/08964289.1990.9934597

[19] Laconi E, Tomasi C, Curreli F, Diana S, Laconi S, et al (2000) Early exposure to restraint stress enhances chemical carcinogenesis in rat liver. Cancer Lett 161: 215–220. 11090972 10.1016/s0304-3835(00)00621-2

[20] Steplewski Z, Vogel WH, Ehya H, Poropatich C, Smith JM (1985) Effects of restraint stress on inoculated tumor growth and immune response in rats. Cancer Res 45: 5128–5133. 3928147

[21] Ben-Eliyahu S, Page GG, Yirmiya R, Shakhar G (1999) Evidence that stress and surgical interventions promote tumor development by suppressing natural killer cell activity. Int J Cancer 80: 880–888. 10074922 10.1002/(sici)1097-0215(19990315)80:6<880::aid-ijc14>3.0.co;2-y

[22] Thaker PH, Han LY, Kamat AA, Arevalo JM, Takahashi R, et al (2006) Chronic stress promotes tumor growth and angiogenesis in a mouse model of ovarian carcinoma. Nat Med 12: 939–944. 16862152 10.1038/nm1447

[23] Schuller HM, Tithof PK, Williams M, Plummer H 3rd (1999) The tobacco-specific carcinogen 4-(methylnitrosamino)-1-(3-pyridyl)-1-butanone is a beta-adrenergic agonist and stimulates DNA synthesis in lung adenocarcinoma via beta-adrenergic receptor-mediated release of arachidonic acid. Cancer Res 59: 4510–4515. 10493497

[24] Weddle DL, Tithoff P, Williams M, Schuller HM (2001) Beta-adrenergic growth regulation of human cancer cell lines derived from pancreatic ductal carcinomas. Carcinogenesis 22: 473–479. 11238189 10.1093/carcin/22.3.473

[25] Schuller HM (2002) Mechanisms of smoking-related lung and pancreatic adenocarcinoma development. Nat Rev Cancer 2: 455–463. 12189387 10.1038/nrc824

[26] Palm D, Lang K, Niggemann B, Drell TLt, Masur K, et al (2006) The norepinephrine-driven metastasis development of PC-3 human prostate cancer cells in BALB/c nude mice is inhibited by beta-blockers. Int J Cancer 118: 2744–2749. 16381019 10.1002/ijc.21723

[27] Wong HP, Yu L, Lam EK, Tai EK, Wu WK, et al (2007) Nicotine promotes colon tumor growth and angiogenesis through beta-adrenergic activation. Toxicol Sci 97: 279–287. 17369603 10.1093/toxsci/kfm060

[28] Zhang D, Ma QY, Hu HT, Zhang M (2010) beta2-adrenergic antagonists suppress pancreatic cancer cell invasion by inhibiting CREB, NFkappaB and AP-1. Cancer Biol Ther 10: 19–29. 20424515 10.4161/cbt.10.1.11944

[29] Rivera CA, Droguett DA, Kemmerling U, Venegas BA (2011) Chronic restraint stress in oral squamous cell carcinoma. J Dent Res 90: 799–803. 21393554 10.1177/0022034511399911

[30] Yin D, Zhang Y, Stuart C, Miao J, Zhang Y, et al (2006) Chronic restraint stress modulates expression of genes in murine spleen. J Neuroimmunol 177: 11–17. 16814870 10.1016/j.jneuroim.2006.05.004

[31] Yin D, Tuthill D, Mufson RA, Shi Y (2000) Chronic restraint stress promotes lymphocyte apoptosis by modulating CD95 expression. J Exp Med 191: 1423–1428. 10770807 10.1084/jem.191.8.1423 PMC2193134

[32] Sheridan JF, Dobbs C, Jung J, Chu X, Konstantinos A, et al (1998) Stress-induced neuroendocrine modulation of viral pathogenesis and immunity. Ann N Y Acad Sci 840: 803–808. 9629306 10.1111/j.1749-6632.1998.tb09618.x PMC1351103

[33] Guo JS, Chau JF, Cho CH, Koo MW (2005) Partial sleep deprivation compromises gastric mucosal integrity in rats. Life Sci 77: 220–229. 15862606 10.1016/j.lfs.2004.12.027

[34] Augsten M, Hagglof C, Olsson E, Stolz C, Tsagozis P, et al (2009) CXCL14 is an autocrine growth factor for fibroblasts and acts as a multi-modal stimulator of prostate tumor growth. Proc Natl Acad Sci U S A 106: 3414–3419. 19218429 10.1073/pnas.0813144106 PMC2651265

[35] Radojcic T, Baird S, Darko D, Smith D, Bulloch K (1991) Changes in beta-adrenergic receptor distribution on immunocytes during differentiation: an analysis of T cells and macrophages. J Neurosci Res 30: 328–335. 1665867 10.1002/jnr.490300208

[36] Abrass CK, O'Connor SW, Scarpace PJ, Abrass IB (1985) Characterization of the beta-adrenergic receptor of the rat peritoneal macrophage. J Immunol 135: 1338–1341. 2409147

[37] Zschauer AO, Sielczak MW, Smith DA, Wanner A (1997) Norepinephrine-induced contraction of isolated rabbit bronchial artery: role of alpha 1- and alpha 2-adrenoceptor activation. J Appl Physiol 82: 1918–1925. 9173959 10.1152/jappl.1997.82.6.1918

[38] Souza RF, Shewmake K, Pearson S, Sarosi GA Jr, Feagins LA, et al (2004) Acid increases proliferation via ERK and p38 MAPK-mediated increases in cyclooxygenase-2 in Barrett's adenocarcinoma cells. Am J Physiol Gastrointest Liver Physiol 287: G743–748. 15231484 10.1152/ajpgi.00144.2004

[39] Tominaga K, Higuchi K, Sasaki E, Suto R, Watanabe T, et al (2004) Correlation of MAP kinases with COX-2 induction differs between MKN45 and HT29 cells. Aliment Pharmacol Ther 20 Suppl 1143–150. 10.1111/j.1365-2036.2004.01986.x 15298620

[40] Shin VY, Wu WK, Chu KM, Koo MW, Wong HP, et al (2007) Functional role of beta-adrenergic receptors in the mitogenic action of nicotine on gastric cancer cells. Toxicol Sci 96: 21–29. 17003101 10.1093/toxsci/kfl118

[41] Calcagno SR, Li S, Colon M, Kreinest PA, Thompson EA, et al (2008) Oncogenic K-ras promotes early carcinogenesis in the mouse proximal colon. Int J Cancer 122: 2462–2470. 18271008 10.1002/ijc.23383 PMC3908548

[42] Haigis KM, Kendall KR, Wang Y, Cheung A, Haigis MC, et al (2008) Differential effects of oncogenic K-Ras and N-Ras on proliferation, differentiation and tumor progression in the colon. Nat Genet 40: 600–608. 18372904 10.1038/ngXXXX PMC2410301

[43] McEwen BS (2000) Allostasis and allostatic load: implications for neuropsychopharmacology. Neuropsychopharmacology 22: 108–124. 10649824 10.1016/S0893-133X(99)00129-3

[44] Glaser R, MacCallum RC, Laskowski BF, Malarkey WB, Sheridan JF, et al (2001) Evidence for a shift in the Th-1 to Th-2 cytokine response associated with chronic stress and aging. J Gerontol A Biol Sci Med Sci 56: M477–482. 11487599 10.1093/gerona/56.8.m477

[45] Page GG, Ben-Eliyahu S (1999) A role for NK cells in greater susceptibility of young rats to metastatic formation. Dev Comp Immunol 23: 87–96. 10220071 10.1016/s0145-305x(98)00040-8

[46] Lutgendorf SK, Sood AK, Anderson B, McGinn S, Maiseri H, et al (2005) Social support, psychological distress, and natural killer cell activity in ovarian cancer. J Clin Oncol 23: 7105–7113. 16192594 10.1200/JCO.2005.10.015

[47] Andersen BL, Farrar WB, Golden-Kreutz DM, Glaser R, Emery CF, et al (2004) Psychological, behavioral, and immune changes after a psychological intervention: a clinical trial. J Clin Oncol 22: 3570–3580. 15337807 10.1200/JCO.2004.06.030 PMC2168591

[48] Lee JW, Shahzad MM, Lin YG, Armaiz-Pena G, Mangala LS, et al (2009) Surgical stress promotes tumor growth in ovarian carcinoma. Clin Cancer Res 15: 2695–2702. 19351748 10.1158/1078-0432.CCR-08-2966 PMC2746852

[49] Tsuchiya Y, Sawada S, Yoshioka I, Ohashi Y, Matsuo M, et al (2003) Increased surgical stress promotes tumor metastasis. Surgery 133: 547–555. 12773983 10.1067/msy.2003.141

[50] Liu H, Wang Z (2005) Effects of social isolation stress on immune response and survival time of mouse with liver cancer. World J Gastroenterol 11: 5902–5904. 16270407 10.3748/wjg.v11.i37.5902 PMC4479698

[51] Armaiz-Pena GN (2008) ((2008)) Chronic stress promotes tumor growth through a Src-dependent mechanism in a mouse model of ovarian cancer. Brain, Behavior, and Immunity 22 8–14.

[52] Shahzad MM, Arevalo JM, Armaiz-Pena GN, Lu C, Stone RL, et al (2010) Stress Effects on FosB- and Interleukin-8 (IL8)-driven Ovarian Cancer Growth and Metastasis. J Biol Chem 285: 35462–35470. 20826776 10.1074/jbc.M110.109579 PMC2975170

[53] Basu S, Nagy JA, Pal S, Vasile E, Eckelhoefer IA, et al (2001) The neurotransmitter dopamine inhibits angiogenesis induced by vascular permeability factor/vascular endothelial growth factor. Nat Med 7: 569–574. 11329058 10.1038/87895

[54] Lara HE, Dorfman M, Venegas M, Luza SM, Luna SL, et al (2002) Changes in sympathetic nerve activity of the mammalian ovary during a normal estrous cycle and in polycystic ovary syndrome: Studies on norepinephrine release. Microsc Res Tech 59: 495–502. 12467025 10.1002/jemt.10229

[55] Lara HE, Porcile A, Espinoza J, Romero C, Luza SM, et al (2001) Release of norepinephrine from human ovary: coupling to steroidogenic response. Endocrine 15: 187–192. 11720244 10.1385/ENDO:15:2:187

[56] Mravec B, Tillinger A, Bodnar I, Nagy GM, Palkovits M, et al (2008) The response of plasma catecholamines in rats simultaneously exposed to immobilization and painful stimuli. Ann N Y Acad Sci 1148: 196–200. 19120109 10.1196/annals.1410.010

[57] Wong HP, Li ZJ, Shin VY, Tai EK, Wu WK, et al (2009) Effects of Cigarette Smoking and Restraint Stress on Human Colon Tumor Growth in Mice. Digestion 80: 209–214. 19776585 10.1159/000231898

[58] Leshan L (1959) Psychological states as factors in the development of malignant disease: a critical review. J Natl Cancer Inst 22: 1–18. 13621196

[59] Frick LR, Rapanelli M, Bussmann UA, Klecha AJ, Arcos ML, et al (2009) Involvement of thyroid hormones in the alterations of T-cell immunity and tumor progression induced by chronic stress. Biol Psychiatry 65: 935–942. 19167703 10.1016/j.biopsych.2008.12.013

[60] Moreno-Smith M, Lu C, Shahzad MM, Pena GN, Allen JK, et al (2011) Dopamine blocks stress-mediated ovarian carcinoma growth. Clin Cancer Res 17: 3649–3659. 21531818 10.1158/1078-0432.CCR-10-2441 PMC3107884

[61] Teunis MA, Kavelaars A, Voest E, Bakker JM, Ellenbroek BA, et al (2002) Reduced tumor growth, experimental metastasis formation, and angiogenesis in rats with a hyperreactive dopaminergic system. Faseb J 16: 1465–1467. 12205050 10.1096/fj.02-0145fje

[62] Askari MD, Tsao MS, Schuller HM (2005) The tobacco-specific carcinogen, 4-(methylnitrosamino)-1-(3-pyridyl)-1-butanone stimulates proliferation of immortalized human pancreatic duct epithelia through beta-adrenergic transactivation of EGF receptors. J Cancer Res Clin Oncol 131: 639–648. 16091975 10.1007/s00432-005-0002-7 PMC12161179

[63] Hasegawa H, Saiki I (2002) Psychosocial stress augments tumor development through beta-adrenergic activation in mice. Jpn J Cancer Res 93: 729–735. 12149137 10.1111/j.1349-7006.2002.tb01313.x PMC5927068

[64] Al-Wadei HA, Al-Wadei MH, Schuller HM (2009) Prevention of pancreatic cancer by the beta-blocker propranolol. Anticancer Drugs 20: 477–482. 19387337 10.1097/CAD.0b013e32832bd1e3 PMC3366433

[65] Shin VY, Jin HC, Ng EK, Yu J, Leung WK, et al (2008) Nicotine and 4-(methylnitrosamino)-1-(3-pyridyl)-1-butanone induce cyclooxygenase-2 activity in human gastric cancer cells: Involvement of nicotinic acetylcholine receptor (nAChR) and beta-adrenergic receptor signaling pathways. Toxicol Appl Pharmacol 233: 254–261. 18805435 10.1016/j.taap.2008.08.012

[66] Zhang D, Ma Q, Shen S, Hu H (2009) Inhibition of pancreatic cancer cell proliferation by propranolol occurs through apoptosis induction: the study of beta-adrenoceptor antagonist's anticancer effect in pancreatic cancer cell. Pancreas 38: 94–100. 19106745 10.1097/MPA.0b013e318184f50c

[67] Slotkin TA, Zhang J, Dancel R, Garcia SJ, Willis C, et al (2000) Beta-adrenoceptor signaling and its control of cell replication in MDA-MB-231 human breast cancer cells. Breast Cancer Res Treat 60: 153–166. 10845278 10.1023/a:1006338232150

[68] Mikami M, Goubaeva F, Song JH, Lee HT, Yang J (2008) beta-Adrenoceptor blockers protect against staurosporine-induced apoptosis in SH-SY5Y neuroblastoma cells. Eur J Pharmacol 589: 14–21. 18534571 10.1016/j.ejphar.2008.04.045 PMC2529477

[69] Schuller HM, Porter B, Riechert A (2000) Beta-adrenergic modulation of NNK-induced lung carcinogenesis in hamsters. J Cancer Res Clin Oncol 126: 624–630. 11079726 10.1007/PL00008474 PMC12165142

[70] Wu WK, Wong HP, Luo SW, Chan K, Huang FY, et al (2005) 4-(Methylnitrosamino)-1-(3-pyridyl)-1-butanone from cigarette smoke stimulates colon cancer growth via beta-adrenoceptors. Cancer Res 65: 5272–5277. 15958573 10.1158/0008-5472.CAN-05-0205

[71] Wong HP, Yu L, Lam EK, Tai EK, Wu WK, et al (2007) Nicotine promotes cell proliferation via alpha7-nicotinic acetylcholine receptor and catecholamine-synthesizing enzymes-mediated pathway in human colon adenocarcinoma HT-29 cells. Toxicol Appl Pharmacol 221: 261–267. 17498763 10.1016/j.taap.2007.04.002

[72] Seya Y, Fukuda T, Isobe K, Kawakami Y, Takekoshi K (2006) Effect of norepinephrine on RhoA, MAP kinase, proliferation and VEGF expression in human umbilical vein endothelial cells. Eur J Pharmacol 553: 54–60. 17070516 10.1016/j.ejphar.2006.09.048

[73] Kim MO, Na SI, Lee MY, Heo JS, Han HJ (2008) Epinephrine increases DNA synthesis via ERK1/2s through cAMP, Ca(2+)/PKC, and PI3K/Akt signaling pathways in mouse embryonic stem cells. J Cell Biochem 104: 1407–1420. 18275042 10.1002/jcb.21716

[74] Dhillon AS, Hagan S, Rath O, Kolch W (2007) MAP kinase signalling pathways in cancer. Oncogene 26: 3279–3290. 17496922 10.1038/sj.onc.1210421

[75] Ruscica M, Dozio E, Motta M, Magni P (2007) Modulatory actions of neuropeptide Y on prostate cancer growth: role of MAP kinase/ERK 1/2 activation. Adv Exp Med Biol 604: 96–100. 17695723 10.1007/978-0-387-69116-9_7

