# Supplementary figures and images for: Effect of Chronic Restraint Stress on Human Colorectal Carcinoma Growth in Mice
Source: PLoS One. 2013 Apr 9;8(4):e61435. doi: 10.1371/journal.pone.0061435 (PMC3621827; doi:10.1371/journal.pone.0061435)

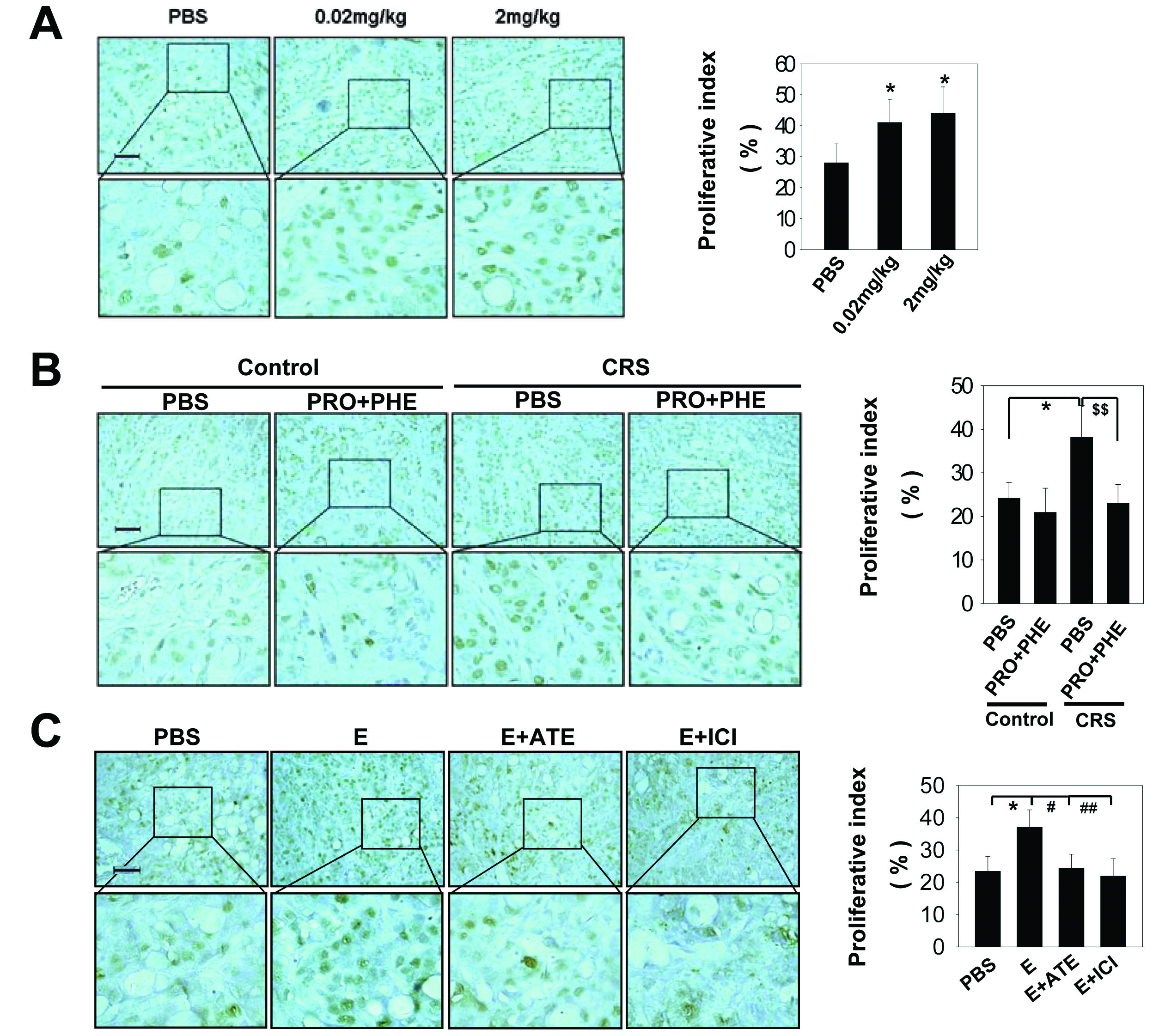

Supplement: Figure S1 — Immunohistochemical staining for PCNA. Immunohistochemical staining for PCNA was performed on (A) HT29 tumor samples from the PBS, 0.02 mg/kg E and 2 mg/kg E treatment groups; (B) HT29 tumor samples from the mice groups treated with PBS or PHE (α-AR antagonist) plus PRO (β-AR antagonist) under CRS or no stress; and (C) HT29 tumor samples from the mice groups treated with PBS, E (0.02 mg/kg), E plus ATE (β1-AR antagonist, 5 mg/kg), or E plus ICI (β2-AR antagonist, 5 mg/kg). Representative tumor sections (left upper panel) and high magnification images of selective portions (left lower panel) from each group are shown. The quantitative data in the graph correspond to the left-side images (right panel). Under microscopy, a dark brown color indicates strong positive immunostaining. Quantified values represent the average immunostaining intensities from at least five random fields of each slide from each tumor tissue, and three to five random tumor tissues from each treatment group are included (magnification: ×400). (Scale bar 50 µm). The data were mean ± SD. * P<0.05, significantly different from the PBS control group; $$ P<0.01 significantly different from the CRS group and # P<0.05, ## P<0.01significantly different from the E-treated group. (TIF) [file pone.0061435.s001.tif]

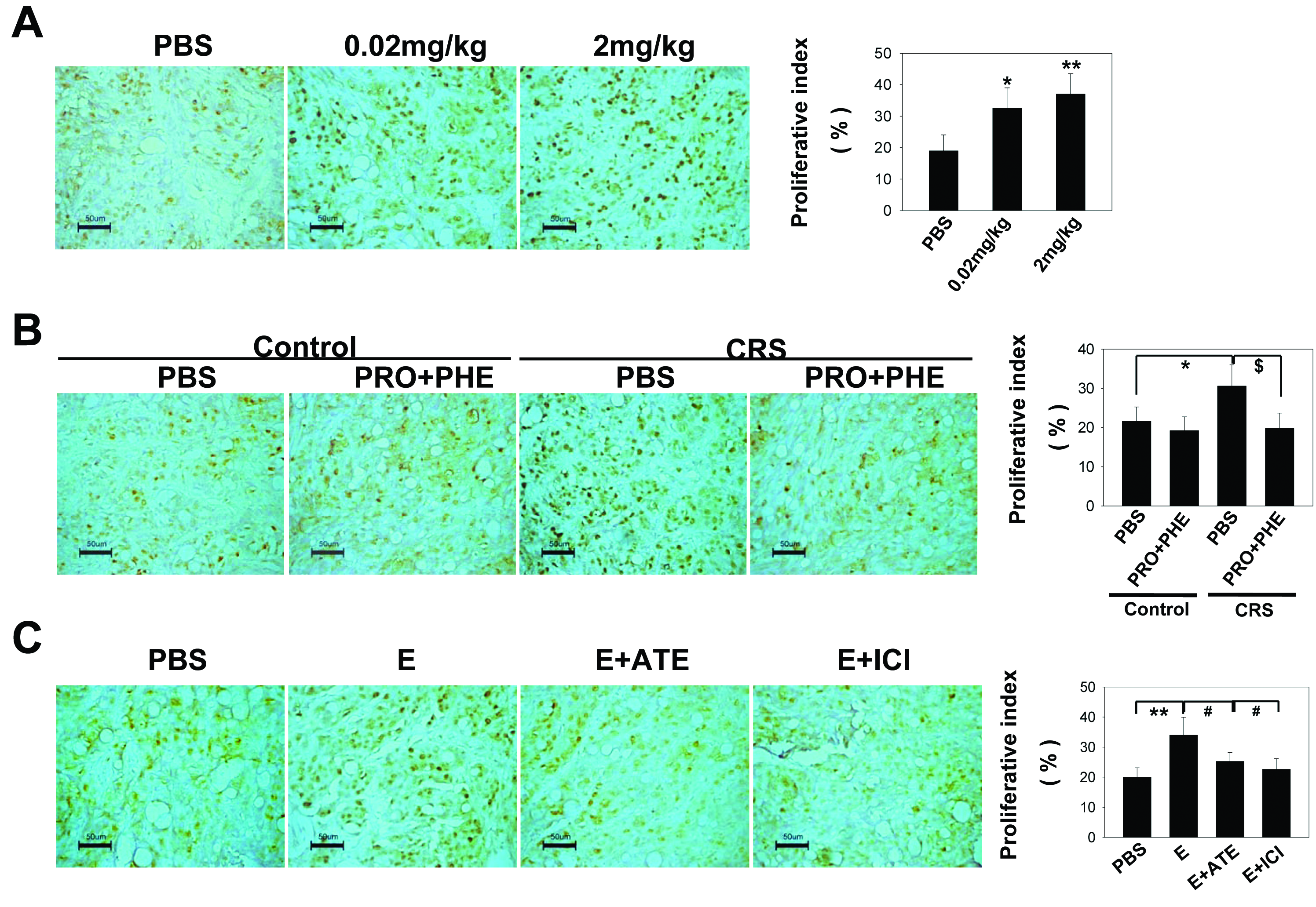

Supplement: Figure S2 — Immunohistochemical staining for Ki-67. Immunohistochemical staining for ki-67 was performed on (A) HT29 tumor samples from PBS, 0.02 mg/kg and 2 mg/kg E treatment groups, (B) HT29 tumor samples from PBS, combination of α-AR antagonist PHE with β-AR antagonist PRO treatments under no-stress or CRS, respectively, and (C) HT29 tumor samples from PBS (used as control), E (0.02 mg/kg), E combined with β1-AR antagonist ATE (5 mg/kg), E combined with β2-AR antagonist ICI (5 mg/kg) treatments were subjected to immunohistochemical staining for Ki-67. Illustrated from each group were representative tumor sections (left panel). The quantitative data in the graph correspond to the left images were shown (right panel). Under microscopy, dark brown color indicates strong positive immunostaining. Quantified values shown were the average immunostaining intensity counted in at least five random fields of each slide from each tumor tissue, and three to five random tumor tissues from each treatment group were included, magnification (×400). (Scale bar 50 µm). The data were mean ± SD. * P<0.05, ** P<0.01, significantly different from the no-stress control group; $ P<0.05 significantly different from the CRS group and # P<0.05 significantly different from the E-treated group. (TIF) [file pone.0061435.s002.tif]

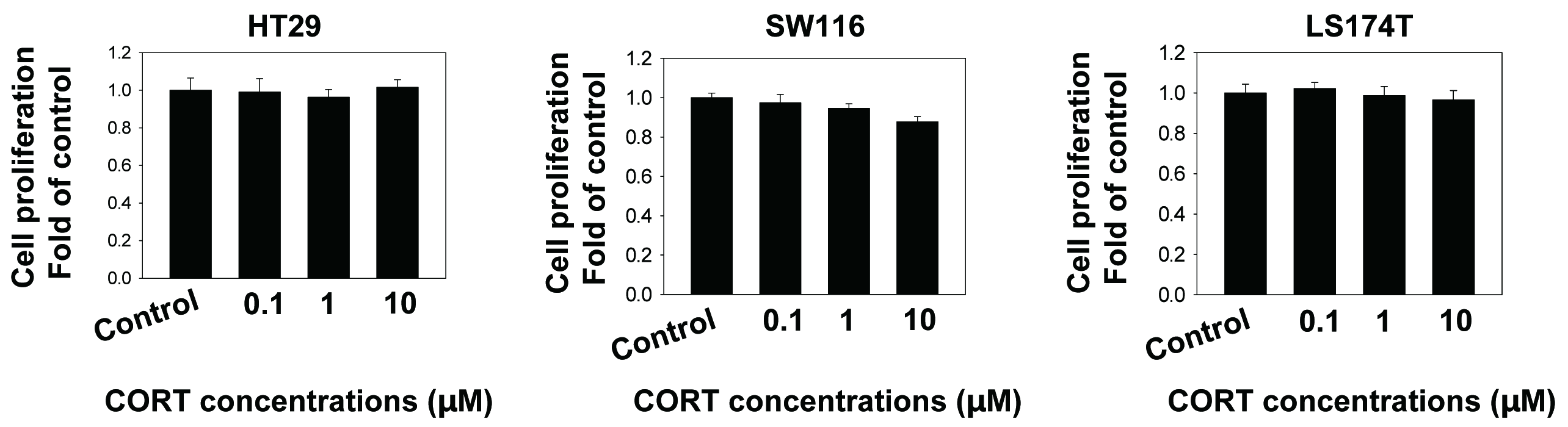

Supplement: Figure S4 — Effects of corticosterone on CRC cells proliferation. CRC HT29, SW116 and LS174T cell lines were treated with different concentrations of corticosterone, as indicated for 24 h, respectively, cell proliferation was measured by BrdU incorporation assay, as described in the materials and methods section. No significant difference was found in any cell line. Data are expressed as mean ± SD of one representative of at least three experiments. (TIF) [file pone.0061435.s004.tif]

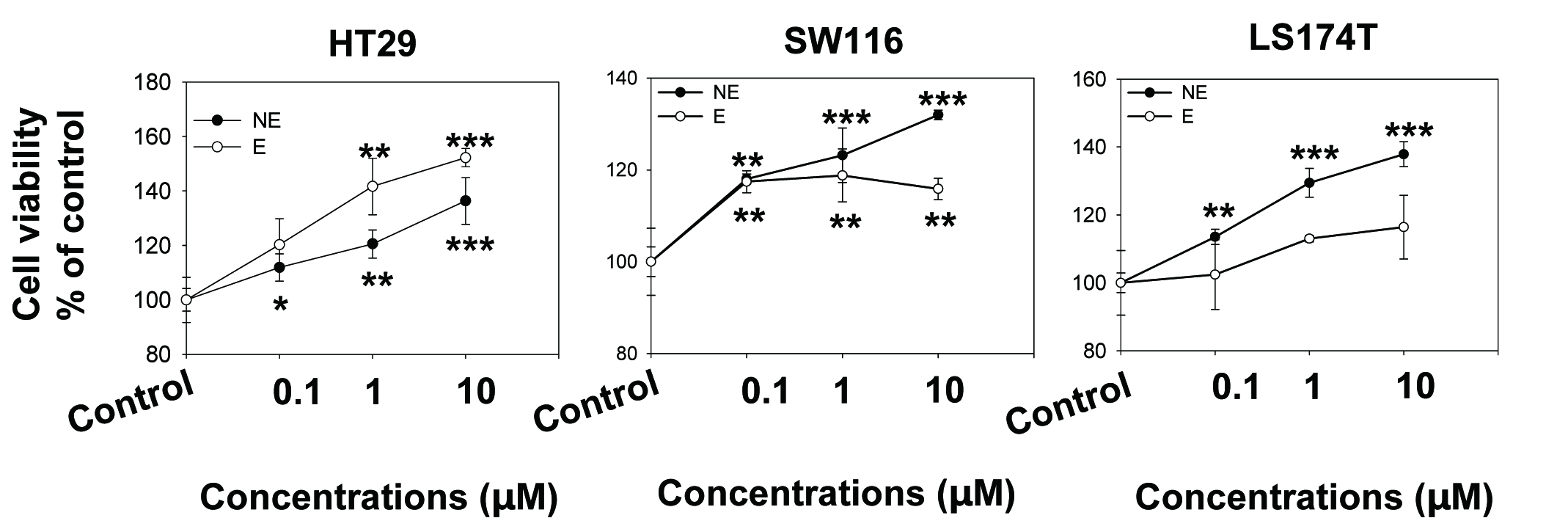

Supplement: Figure S5 — Effects of E or NE on CRC cells viability. The CRC HT29, SW116 and LS174T cell lines were seeded in 96-well plates and cultured in the presence of different concentrations of E or NE for 24 h, as indicated. Cell viability was measured by CCK-8 assay, as described in the Materials and Methods section. Both E and NE significantly promoted all three CRC cell lines survival in a dose-dependent manner. The results are expressed as mean ± SD of one representative of three independent experiments. * P<0.05, ** P<0.01, *** P<0.001 significantly different from the control group. (TIF) [file pone.0061435.s005.tif]

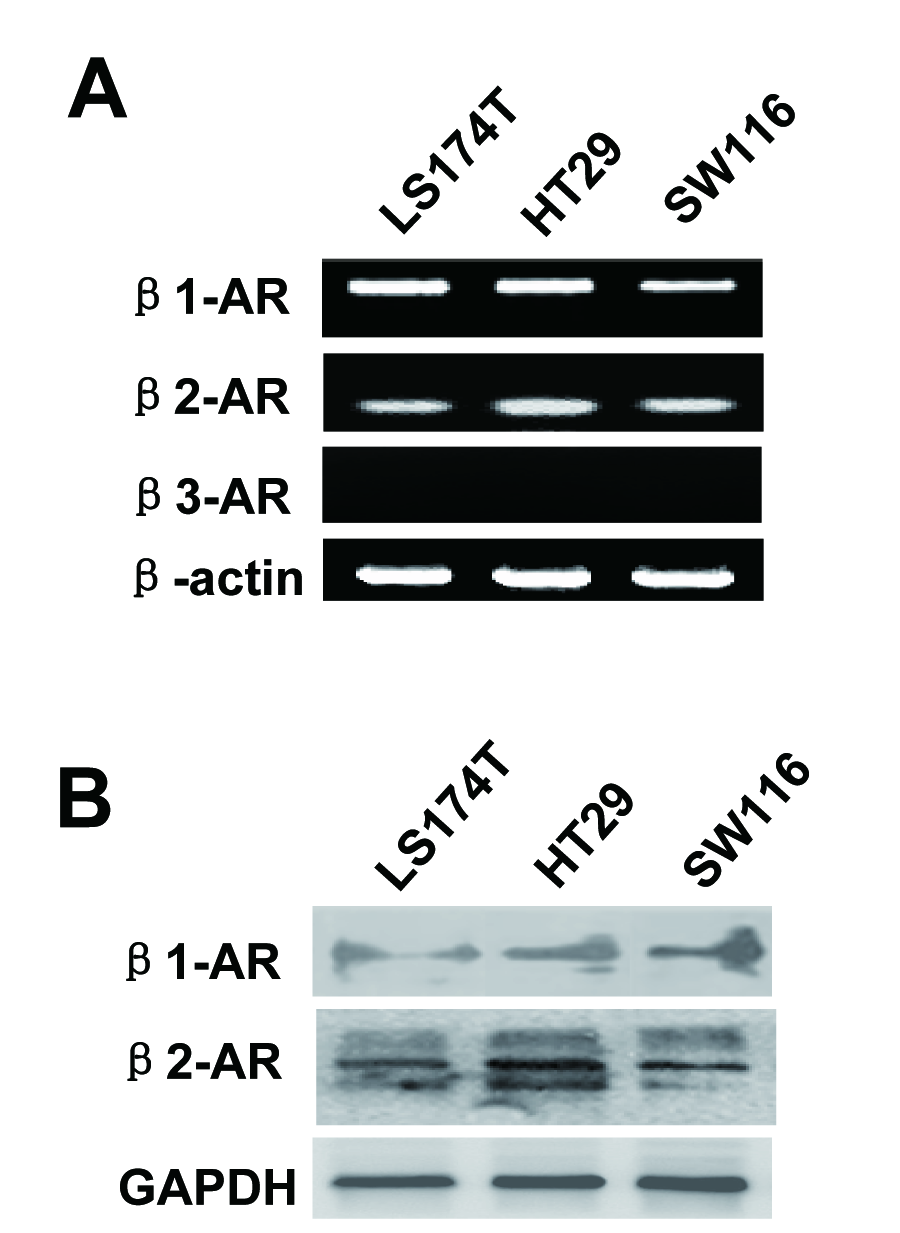

Supplement: Figure S6 — Expression of β1- and β2-AR in CRC cell lines. (A) RT-PCR was performed to determined β1-, β2-AR and β3-AR mRNA levels in CRC HT29, SW116 and LS174T cells. Representative RT-PCR assay were shown. (B) Lysates from CRC HT29, SW116 and LS174T cell lines using specific monoclonal antibody probed for β1-AR and polyclonal antibody probed for β2-AR. Both β1- and β2-AR protein expressed in CRC HT29, SW116 and LS174T cell lines by western blot analysis. (TIF) [file pone.0061435.s006.tif]

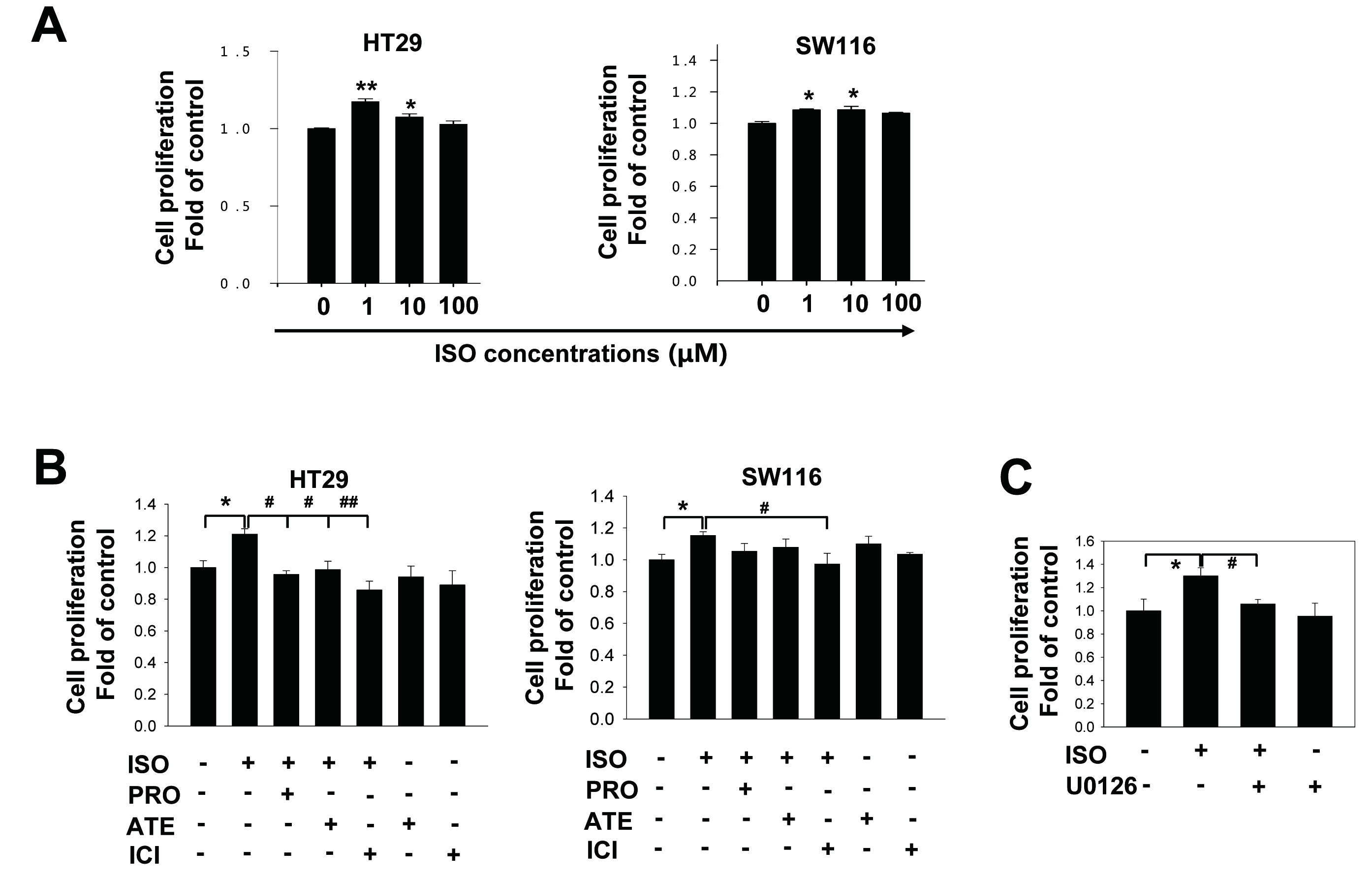

Supplement: Figure S7 — Involvement of ERK1/2 in the β-AR-mediated CRC cells proliferation. (A) HT29 (left panel) and SW116 cells (right panel) were treated with different concentrations of β-AR agonist ISO, as indicated for 24 h, respectively. Result of BrdU incorporation assays showed ISO remarkably induced both CRC cells proliferation at dose of 1 µM. Data are expressed as mean ± SD of one representative of three experiments. * P<0.05, ** P<0.01 significantly different from the control group. (B) HT29 (left panel) and SW116 cells (middle panel) were pretreated with β-AR antagonist PRO, β1-AR antagonist ATE (50 µM) or β2-AR antagonist ICI (50 µM) for 45 min before incubation with β-AR agonist ISO (1 µM), respectively. After 24 h, cell proliferation was measured by BrdU incorporation assay, as described in the materials and methods section. Data were expressed as mean ± SD of triplicate or quadruplicate samples per treatment group in at least three independent experiments with similar results. * P<0.05 significantly different from the control group and # P<0.05, ## P<0.01, significantly different from the ISO-treated group, (C)HT29 cells were pretreated with or without ERK1/2 specific inhibitor U0126 (20 µM) for 45 min before incubation with ISO (1 µM). Results showed that ISO-induced cell proliferation was remarkably blocked by ERK1/2 specific inhibitor U0126. Data were expressed as mean ± SD of triplicate or quadruplicate samples per treatment group in at least three independent experiments with similar results. * P<0.05, significantly different from the control group and # P<0.05, significantly different from the ISO-treated group. (TIF) [file pone.0061435.s007.tif]
